# Practices and Perceptions of Infectious Diseases and Critical Care Clinicians Regarding Aerosolized Antimicrobial Therapy in the State of Qatar: A Multicenter Cross-Sectional Study

**DOI:** 10.3390/antibiotics15090882

**Published:** 2026-09-09

**Authors:** Noran Magdy Zahran, Usman Abubakar, Asmaa Ezzeldin Mohd. Mohamed, Hani Jaouni, Sayed Tarique Kazi, Muna Al-Maslamani

**Affiliations:** 1 Department of Clinical Pharmacy and Practice, College of Pharmacy, QU Health, Qatar University, Doha P.O. Box 2713, Qatar; 2 Department of Pharmacy, Hamad Medical Corporation, Doha P.O. Box 3050, Qatar; 3 Critical Care Department, Hamad General Hospital, Hamad Medical Corporation, Doha P.O. Box 3050, Qatar; 4 College of Medicine, QU Health, Qatar University, Doha P.O. Box 2713, Qatar; 5 Critical Care Department, Al-Wakra Hospital, Hamad Medical Corporation, Doha P.O. Box 3050, Qatar; 6 Division of Infectious Diseases, Department of Medicine, Hamad Medical Corporation, Doha P.O. Box 3050, Qatar; 7 Communicable Disease Center, Hamad Medical Corporation, Doha P.O. Box 3050, Qatar

**Keywords:** aerosolized antimicrobial therapy, prescribing, perception, practices, infectious diseases, critical care, cross-sectional study, Qatar

## Abstract

**Background/Objectives**: The prescription of aerosolized antimicrobial agents for lower respiratory tract infections varies significantly among clinicians. This study investigated the practices and perceptions of clinicians regarding aerosolized antimicrobial therapy. **Methods:** A cross-sectional study was conducted among infectious diseases (ID) and critical care clinicians (physicians and clinical pharmacists) in eight acute care hospitals in Qatar using a validated and pretested self-administered online questionnaire. Data was collected between September and October 2025, and analyzed using the Statistical Package for the Social Sciences (SPSS). **Results:** The majority of the respondents were physicians (70.5%), and about one-third (31.8%) had more than 15 years of work experience. A vast majority (97.7%) had previously prescribed or recommended aerosolized antimicrobial agents. Some respondents (34.1%) reported using aerosolized antimicrobial agents at least once every three months. The indications for aerosolized antimicrobial prescribing were for the treatment of bacterial infections (86.4%) and prophylaxis against bacterial infections (38.6%). Aerosolized antimicrobials were used for the treatment of ventilator-associated pneumonia (68.2%), cystic fibrosis (61.4%) and ventilator-associated tracheobronchitis (34.1%). Gentamicin (88.6%) and colistin (72.7%) were the most frequently used aerosolized antimicrobial agents. The majority (88.6%) of the respondents combined aerosolized antimicrobial agents with systemic antimicrobial agents, 65.9% of whom combined different antimicrobials for aerosolized and systemic routes. *Pseudomonas aeruginosa* (97.7%) and *Acinetobacter baumannii* (56.8%) were the most common microorganisms treated with aerosolized antimicrobial agents. Most respondents (61.4%) did not provide pre-treatment prior to aerosolized antimicrobial therapy. However, 38.6% used bronchodilators for pre-treatment. Bronchospasm (79.5%) and hypoxemia (20.5%) were the adverse effects reported by the respondents. Some respondents (40.9%) strongly agreed/agreed that aerosolized antimicrobial therapy improves clinical and microbial cure rates. While 81.8% strongly agreed/agreed that aerosolized antimicrobial therapy reduces systemic toxicity. Practices and perceptions varied significantly between ID and critical care specialties, and between physicians and pharmacists. **Conclusions:** Clinicians need training on aerosolized antimicrobial therapy for lower respiratory tract infections. Immunosuppression, renal function and infections due to multidrug-resistant organisms were reported by clinicians as factors that influenced their decision to use aerosolized antimicrobial therapy. A protocol for the administration of aerosolized antimicrobial therapy is recommended.

## 1. Introduction

Lower respiratory tract infections (LRTIs), including hospital-acquired pneumonia, ventilator-associated pneumonia (VAP) and ventilator-associated tracheobronchitis (VAT), are common nosocomial infections among critically ill patients, and they are associated with morbidity, mortality, and excessive healthcare costs [1,2]. A central challenge in the management of LRTIs in the ICU is the relentless rise in multidrug-resistant (MDR) Gram-negative pathogens, particularly *Pseudomonas aeruginosa*, *Acinetobacter baumannii*, and Enterobacterales [2,3]. These organisms have progressively eroded the therapeutic arsenal, leaving clinicians with few reliable systemic options [4]. Conventional intravenous antibiotics often fail to achieve adequate pulmonary concentrations against such pathogens, particularly when minimum inhibitory concentrations are elevated, leaving clinicians in urgent need of alternative drug delivery strategies [4].

Aerosolized antimicrobial therapy has emerged in this context as a biologically compelling approach. By delivering drugs directly to the site of infection via inhalation, nebulized antibiotics can achieve local concentrations that far exceed what could be safely attained through systemic administration, while simultaneously limiting exposure and thus toxicity at peripheral sites [1,3]. These pharmacokinetic advantages are particularly meaningful for agents with narrow therapeutic indices, such as aminoglycosides and polymyxins, which are often used against MDR pathogens [4,5]. Recent advances in nebulizer technology and aerosol delivery systems have renewed interest in evidence-based aerosolized therapy [1,6,7]. A 2024 systematic review and meta-analysis reported that adjunctive inhaled colistin or tobramycin may improve clinical and microbiological outcomes in patients with VAP caused by Gram-negative bacteria [8]. Another review reported that while several randomized controlled trials have explored inhaled amikacin, tobramycin, colistin, and ceftazidime for VAP with some demonstrating improved clinical cure rates and shorter ICU stays, there are no definitive conclusions on patient-centered outcomes due to variability across study designs and populations [2]. A meta-analysis of randomized controlled trials further confirmed that prophylactic inhaled antibiotics, particularly aminoglycosides such as amikacin, significantly reduced VAP incidence, but their impact on mortality, ICU length of stay, and duration of mechanical ventilation remained statistically inconclusive [9]. Aerosolized antimicrobial therapy has also demonstrated clinical benefit in chronic respiratory diseases such as bronchiectasis, further establishing its therapeutic plausibility [10].

Aerosolized antimicrobial therapy faces several practical and scientific challenges, including significant variability in nebulizer device selection, ventilator circuit configurations, drug formulations, particle size and pulmonary deposition, and administration techniques. These challenges affect drug delivery and clinical outcomes [1,7]. In addition, concerns about bronchospasm, ventilator circuit obstruction, and the potential for selecting resistant organisms have been raised [4,5]. Furthermore, the pharmacokinetic and pharmacodynamic data needed to guide rational inhaled antibiotic dosing in critically ill patients are limited, leaving practitioners to prescribe in an environment of considerable uncertainty [5]. Current international guidelines reflect this uncertainty with deliberately cautious recommendations. The 2016 Infectious Diseases Society of America (IDSA) and American Thoracic Society (ATS) guidelines advocate adjunctive inhaled antibiotics only in selected patients with VAP caused by Gram-negative bacilli susceptible solely to aminoglycosides or polymyxins [11]. The European Society of Clinical Microbiology and Infectious Diseases (ESCMID) similarly restricts its endorsement of aerosolized antimicrobial therapy to carefully defined clinical scenarios, citing insufficient high-quality evidence to support routine use [12]. In spite of these cautious recommendations, clinicians worldwide continue to prescribe nebulized antibiotics, often outside guideline boundaries, driven by the grim realities of MDR infections and a perceived lack of viable alternatives [13,14,15]. International surveys have consistently documented striking heterogeneity in how clinicians approach aerosolized antimicrobial therapy across institutions and countries [13,14,15]. Understanding the drivers of this variability is essential to identify educational needs and to guide protocol development for consistent, evidence-informed practice [13]. In Qatar, antimicrobial resistance remains a healthcare challenge, particularly within the ICU setting where MDR Gram-negative infections are disproportionately common [16]. Currently, there is a lack of data to describe how infectious diseases physicians and critical care clinicians in Qatar prescribe aerosolized antimicrobial agents. Therefore, this multicenter cross-sectional study aims to evaluate the current practices and perceptions of infectious diseases and critical care clinicians regarding aerosolized antimicrobial therapy in Qatar and to identify the key factors that shape prescribing behaviors in this population.

## 2. Results

### 2.1. Qualification and Practice-Related Information of the Respondents

Forty-six respondents completed the questionnaire (response rate: 35.4%), with two ineligible responses (one emergency department clinical pharmacist and one hospital epidemiologist) excluded. In total, 44 responses were included in the analysis, the majority of which were from physicians (70.5%). Approximately one-third (31.8%) of the respondents had more than 15 years of work experience. Critical care physicians (consultants/specialists/residents) accounted for 50% of the respondents, followed by infectious diseases (ID)/antimicrobial stewardship (AMS) pharmacists (22.7%). Overall, there was at least one response from eight acute care hospitals in Qatar. Table 1 summarizes the qualifications and practice-related information of the respondents.

### 2.2. Clinicians’ Practices Regarding Aerosolized Antimicrobial Agents

#### 2.2.1. Frequency, Types and Indications for Aerosolized Antimicrobial Therapy

A vast majority of the respondents had previously prescribed or recommended aerosolized antimicrobial agents for their patients. The frequency of aerosolized antimicrobial therapy varied, with 34.1% using aerosolized antimicrobials at least once in three months, followed by “at least once in six months” (22.7%) and “at least once in a month” (18.2%). The treatment of bacterial infections was the most common indication for aerosolized antimicrobial agents (86.4%), followed by prophylaxis against bacterial infections (38.6%) and prophylaxis against fungal infections (15.9%). Prophylaxis against bacterial infections was significantly higher among ID clinicians (57.9%) compared with critical care clinicians (24.0%). Prophylaxis against fungal infections was more common among pharmacists compared with physicians (38.5% versus 6.5%; *p* = 0.017). Respondents indicated that aerosolized antimicrobials were commonly used for ventilator-associated pneumonia (68.2%), cystic fibrosis (61.4%), ventilator-associated tracheobronchitis (34.1%), and hospital-acquired pneumonia (29.5%) (Table 2). ID clinicians indicated higher use of aerosolized antimicrobial agents for cystic fibrosis (89.5%) than critical care clinicians did (40.0%); *p* = 0.001. Gentamicin (88.6%) and colistin (72.7%) were the most frequently used aerosolized antimicrobial agents. Other agents used by the respondents included amikacin (25.0%), tobramycin (15.9%), and amphotericin B (9.1%) (Table 2).

#### 2.2.2. Treatment and Administration Strategies Used for Aerosolized Antimicrobial Therapy

The majority of the respondents combined aerosolized antimicrobial agents with systemic antimicrobial agents (88.6%), and about two-thirds (65.9%) of them combined different antimicrobials for aerosolized and systemic administrations. Respondents indicated that *Pseudomonas aeruginosa* (97.7%) and *Acinetobacter baumannii* (56.8%) were the most common microorganisms they treated with aerosolized antimicrobial agents. Approximately 80% of the respondents indicated that multidrug resistance or carbapenem resistance influenced their decision to use aerosolized antimicrobial agents. Some respondents (43.2%) were unaware of the aerosolization device used in their hospital. However, 31.8% reported using a jet nebulizer, followed by an ultrasonic nebulizer (18.2%) and a vibrating-mesh nebulizer (15.9%). The majority of the respondents (61.4%) indicated that they do not provide pre-treatments prior to aerosolized antimicrobial therapy. However, 38.6% reported using pre-treatment with a bronchodilator. Patient factors that influenced respondents’ decision to use aerosolized antimicrobial therapy included immunosuppression (72.7%), neutropenia (47.7%), advanced age (45.5%) and chronic kidney disease (43.2%). Critical care clinicians indicated a significantly higher influence of diabetes mellitus on their decision to use aerosolized antimicrobial therapy than ID clinicians did. The most common adverse effects of aerosolized antimicrobial therapy reported by clinicians were bronchospasm (79.5%) and hypoxemia (20.5%). Most of the respondents (56.8%) indicated that their hospital lacked a protocol for aerosolized antimicrobial therapy. Table 3 shows responses for treatment and administration strategies for aerosolized antimicrobial therapy.

### 2.3. Clinicians’ Perception Regarding Aerosolized Antimicrobial Agents

Only one-third of the respondents (35.3%) indicated that they were very/well informed about aerosolized antimicrobial therapy, while the majority (81.9%) highly rated (3–5) their need for aerosolized antimicrobial therapy training. More ID clinicians perceived that they were very well/well informed about aerosolized antimicrobial therapy (52.6%) than critical care clinicians (24.0%). In addition, 40.9% strongly agreed/agreed that aerosolized antimicrobial therapy improves clinical and microbial cure rates. More physicians strongly agreed/agreed that aerosolized antimicrobial therapy improves clinical cure (51.6%) compared with pharmacists (15.4%), *p* = 0.015. In addition, more than one-third of pharmacists (38.5%) strongly disagreed/disagreed that aerosolized antimicrobial therapy improves microbial outcomes as compared with 9.7% of physicians. Approximately 32% of ID clinicians and 8.0% of critical care clinicians strongly disagreed/disagreed that aerosolized antimicrobial therapy improves microbial outcomes. While the majority of the respondents (81.8%) strongly agreed/agreed that aerosolized antimicrobial therapy reduces systemic toxicity, only 36.4% strongly agreed/agreed that aerosolized antimicrobial therapy allows for the administration of higher doses of antimicrobial agents, with more pharmacists (53.8%) indicating strong agreement/agreement than physicians (29.0%); *p* = 0.045. Approximately 41% of the respondents were neutral regarding the statement “aerosolized antimicrobial therapy can reduce antimicrobial resistance,” and 29.5% indicated strong agreement/agreement with the same statement. The most common barrier to the use of aerosolized antimicrobial therapy was limited evidence on efficacy (88.6%), followed by the availability of medications (34.1%) and limited evidence on safety (20.5%). Table 4 presents the perceptions of the respondents toward aerosolized antimicrobial therapy.

## 3. Discussion

The current study evaluated the practices and perceptions of ID and critical care clinicians toward aerosolized antimicrobial therapy in multiple hospitals in the State of Qatar. The results revealed that aerosolized antimicrobial therapy is common, as almost all the respondents had previously used aerosolized antimicrobial agents on their patients. This is consistent with the findings of a previous international survey where nebulization of antimicrobials was a common treatment modality for respiratory infections among intensive care unit patients [14]. In addition, a considerable proportion of the clinicians reported prescribing or recommending aerosolized antimicrobial therapy quarterly. The results of the current study showed that aerosolized antimicrobial agents are commonly used for the treatment of and prophylaxis against bacterial infections, and this is in consonance with the findings of a previous study [14]. Evidence has proven that aerosolized antibiotics are beneficial in preventing pneumonia in critically ill patients on mechanical ventilation [17,18]. While aerosolized antibiotics improve microbiological eradication in VAP, their benefit in improving clinical outcomes has been inconsistent [8,19,20,21]. VAP, cystic fibrosis and VAT were the most common indications for aerosolized antibiotics in the current study, and this is consistent with the result of a global survey [13,14]. Consistent with prior studies [13,14], nephrotoxic antimicrobials, namely aminoglycosides (gentamicin, amikacin and tobramycin), colistin and amphotericin B, were the most common antimicrobial agents prescribed for aerosolized antimicrobial therapy. Parenteral aminoglycosides achieve poor concentrations in the lung tissue due to their limited bronchial penetration [22]. While higher concentrations of aminoglycosides and colistin are required for the eradication of multidrug-resistant Gram-negative organisms from the lungs, potential nephrotoxicity from higher systemic doses is a concern, thereby prompting the need for adjunctive aerosolized therapy. Experimental studies have shown that aerosolized antibiotics achieved higher concentrations in the epithelial lining fluid compared with systemic antibiotics [4,22,23], and this correlates with positive clinical outcomes in VAP patients [23].

The vast majority of the participants used aerosolized antibiotics in combination with systemic ones. Clinical studies support this practice due to the clinical and microbiological benefits associated with the combination of aerosolized and systemic antimicrobial agents [8,19,24,25,26]. Most participants reported the combination of different antimicrobials for systemic and aerosolized administration. Comparative studies reporting the efficacy and safety of different antimicrobial combinations for systemic and aerosolized administration over the use of the same antimicrobial agent for both routes of administration are currently lacking. However, the combination of different antimicrobials could be explained by the need to overcome antimicrobial resistance using antimicrobials with different mechanisms of action. Globally, the treatment of respiratory tract infections caused by multidrug-resistant Gram-negative bacilli is an indication for aerosolized antimicrobial therapy [14]. This is due to the poor concentrations of antimicrobials in the lung tissue, coupled with the need to achieve higher concentrations to eradicate multidrug-resistant Gram-negative bacilli infections in the lungs. The study found that multidrug-resistant Gram-negative bacilli, including *Pseudomonas aeruginosa*, *Acinetobacter baumannii* and Enterobacterales, were the most common organisms treated with aerosolized antimicrobial agents. In addition, more than two-thirds of the respondents indicated that multidrug resistance and carbapenem resistance influenced their decision to use aerosolized antimicrobial agents. Furthermore, immunosuppression, advanced age and chronic kidney disease were also reported to influence clinicians’ decisions regarding aerosolized antimicrobial therapy.

Although aerosolized antimicrobial therapy improves microbiologic eradication and clinical cure rates in some studies [8,19,26], bronchospasm and hypoxemia are common adverse effects associated with this therapy [26,27,28]. These adverse effects associated with aerosolized antimicrobial use explain the prophylactic use of bronchodilators by more than one of clinicians in the current study. However, over 60% provided no pre-treatment for their patients. This highlights an important opportunity for the development of antimicrobial stewardship interventions to improve the use of aerosolized antimicrobial therapy. The participants indicated the lack of a protocol for aerosolized antimicrobial therapy in participating hospitals, consistent with the findings of a global survey [14]. Therefore, a local protocol for aerosolized antimicrobial therapy is required to standardize the treatment approach and improve patient outcomes. The perception of clinicians in the current study toward aerosolized antimicrobial therapy mirrors the results of clinical studies that investigated the efficacy and safety of aerosolized antimicrobial therapy [8,19,20,21,24,25,26]. Some studies have shown that aerosolized antibiotics did not improve clinical and microbiological benefits [20,21,29], whereas other studies revealed otherwise [8,19,25]. In addition, published studies on this subject matter are adjudged to have low quality [8], and this highlights the need for further high-quality studies [8,19,20,22]. Most participants required training on aerosolized antimicrobial therapy, and this represents an opportunity for antimicrobial stewardship teams to provide regular updates on the use of aerosolized antimicrobial agents. The practices and perceptions of clinicians varied significantly based on the specialty and profession in the current study. This observation underlines the need for educational interventions targeted at the needs of different specialties and professions.

There are some limitations that should be considered in the interpretation of the results. First, the low response rate (*n* = 46; 35.4%) limits the generalizability of the findings even though there were responses from eight acute care hospitals in Qatar. In addition, single-respondent percentage shifts are large due to the low response rate. Data was collected over 6 weeks with multiple reminders sent to the participants. Second, the responses were self-reported, and they are susceptible to social desirability bias and recall bias. Third, the study was limited to ID physicians and clinical pharmacists, and critical care physicians and clinical pharmacists only, thereby excluding other healthcare professionals who could provide additional insights into the utilization of aerosolized antimicrobial therapy in the State of Qatar. Future studies should include nurses and respiratory therapists who are also involved in the administration of aerosolized antimicrobial therapy. However, the study provides some insights into the practices and perceptions of clinicians toward aerosolized antimicrobial therapy in Qatar. Future studies should evaluate the prescribing patterns for aerosolized antimicrobial therapy among patients in Qatar and identify opportunities for antimicrobial stewardship interventions.

## 4. Materials and Methods

### 4.1. Study Design

This multicenter cross-sectional study was conducted among infectious diseases (ID) and medical intensive care unit (MICU) clinicians across Hamad Medical Corporation (HMC) hospitals in the State of Qatar.

### 4.2. Study Settings

Acute care hospitals under the HMC, including Hamad General Hospital, Al-Wakra Hospital, Al-Khor Hospital, Hazm Mebaireek General Hospital, Communicable Diseases Center, Rumailah Hospital, The Cuban Hospital, and the Women Wellness and Research Center, were included in the study.

### 4.3. Study Population

Practicing infectious diseases (IDs) and medical intensive care unit (MICU) clinicians across Hamad Medical Corporation (HMC) hospitals were invited to participate in the study. Such healthcare professionals routinely manage patients with lower respiratory tract infections, including ventilator-associated pneumonia (VAP) and ventilator-associated tracheobronchitis (VAT), conditions for which aerosolized antimicrobial therapy may be considered as part of the treatment strategy.

Inclusion Criteria:

- ID consultants, specialists and residents practicing in participating HMC hospitals.
- MICU consultants, specialists and residents practicing in HMC hospitals involved in the study.
- Antimicrobial stewardship (AMS) pharmacists practicing in HMC hospitals involved in the study.
- MICU clinical pharmacists practicing in HMC hospitals involved in the study.
- Those who provide consent to participate in the study.
- Those who can read and write in the English language.

Exclusion Criteria:

- Those who declined to participate in the study.
- Those who did not submit completed responses within the data collection period.

### 4.4. Sample Size Determination

The study population consisted of 73 infectious diseases (IDs) clinicians, including 52 ID physicians and 21 antimicrobial stewardship (AMS) pharmacists, as well as 57 medical intensive care unit (MICU) clinicians, comprising 52 MICU physicians and five MICU pharmacists. Given the relatively small study population, all eligible clinicians were invited to participate in the study.

### 4.5. Questionnaire Development and Validation

A self-administered online questionnaire was used for data collection. Previously published studies were reviewed in the buildup to the questionnaire development [13,14,15]. The questionnaire had three sections and 28 items distributed as follows: qualifications and practice-related information (six items), practices regarding aerosolized antimicrobial therapy (14 items) and perceptions regarding aerosolized antimicrobial therapy (eight items). A panel of three experts with expertise in questionnaire design and one ID clinician who is conversant with aerosolized antimicrobial therapy validated the questionnaire. The panel validated each item for clarity and relevance. The questionnaire was revised based on the comments and suggestions of the reviewers. The item Content Validity Index (I-CVI) ranged from 0.67 to 1.00 for the practices domain and was 1.0 for the perceptions domain. The Scale Content Validity Index for the practices and perception domains was 0.97 and 1.00, respectively. The item Face Validity Index (I-FVI) ranged from 0.33 to 1.00 for the practices domain and was 0.67 for the perceptions domain. Overall, the Scale Face Validity Index for the practices and perceptions domains was 0.83 and 0.67, respectively. The questionnaire was pretested among 10 clinicians to determine the reliability, and the Cronbach’s alpha value was 0.77.

### 4.6. Data Collection

Data was collected using a self-administered, validated and pretested online questionnaire. ID and MICU clinicians (physicians and pharmacists) working in the participating hospitals were invited to the study. The questionnaire was prepared using Microsoft Forms, and the hyperlink to the online questionnaire was distributed to eligible clinicians via email or WhatsApp. The questionnaire had an information and consent section that described the study objectives and requested participants’ consent before proceeding to complete the questionnaire. Data was collected over a 6-week period from September to October 2025.

### 4.7. Data Analysis

Data analysis was performed using the Statistical Package for the Social Sciences (SPSS) software for windows version 30. Descriptive statistics were used to summarize the data. Categorical variables were presented as frequencies and percentages, while continuous variables were summarized using the median and interquartile range (IQR) for non-normally distributed data, and the mean ± standard deviation (SD) for normally distributed data. Inferential statistics using the Chi-Square Test and Fisher’s Exact Test, where applicable, were used to compare responses between the ID and critical care specialties, and between physicians and pharmacists. A *p* value of less than 0.05 was considered statistically significant.

### 4.8. Ethical Consideration

The study protocol was reviewed and approved by the Medical Research Center of Hamad Medical Corporation (reference number: MRC-02-25-345). As all participating hospitals are affiliated with HMC, institutional approval was obtained from the respective hospitals. In addition, ethical approval for the study was obtained from the Qatar University Institutional Review Board (reference number: QU-IRB 310/2024-EM).

## 5. Conclusions

This study showed that aerosolized antimicrobial therapy is frequently used among infectious diseases and critical care clinicians in Qatar, particularly for lower respiratory tract infections caused by multidrug-resistant organisms. Clinicians’ decisions to use aerosolized antimicrobial agents were reportedly influenced by several patient- and infection-related factors, including multidrug resistance, immunosuppression, and renal function. However, differences in practice patterns and the limited availability of institutional protocols indicate a need for greater consistency in the use of this therapy. In addition, the need for further education reported by many clinicians highlights the importance of providing targeted training and strengthening antimicrobial stewardship efforts. Developing evidence-based local protocols may help standardize practice and support the safer and more effective use of aerosolized antimicrobial therapy.

## Figures and Tables

**Table 1:** Respondents’ qualification and practice-related information.

Variable	Frequency (N = 44)	Percentage (%)
Highest qualification		
MD	20	45.5
MBBS	8	18.2
PharmD	7	15.9
MSc.	5	11.4
PhD	2	4.5
Others	2	4.5
Country of graduation		
Qatar	6	13.6
Other countries	38	86.4
Years of experience		
1–5	6	13.6
6–10	12	27.3
11–15	12	27.3
More than 15	14	31.8
Profession		
Physician	31	70.5
Pharmacist	13	29.5
Professional cadre		
Critical Care Physician (Consultant, Specialist, Resident)	22	50.0
ID/AMS Pharmacist	10	22.7
ID Physician (Consultant, Specialist, Resident)	9	20.5
Critical Care Pharmacist	3	6.8
Practice location		
Hamad General Hospital	20	45.5
Al-Wakra Hospital	10	22.7
Communicable Disease Center	5	11.4
Al-Khor Hospital	3	6.8
Cuban Hospital	2	4.5
Rumailah Hospital	2	4.5
Others	2	4.5

MD: Doctor of Medicine; MBBS: Bachelor of Medicine and Bachelor of Surgery; MSc.: Master of Science; PhD: Doctor of Philosophy; ID: infectious diseases; AMS: antimicrobial stewardship.

**Table 2:** Frequency, types and indications for aerosolized antimicrobial therapy among respondents.

		Specialty			Profession		
	Total	Frequency (%)			Frequency (%)		
Variable	Frequency (N = 44)	ID (N = 19)	Critical Care (N = 25)	*p* Value	Physicians (N = 31)	Pharmacists (N = 13)	*p* Value
Have you prescribed/recommended an aerosolized antimicrobial agent before?				1.000			1.000
Yes	43 (97.7)	19 (100)	24 (96.0)		30 (96.8)	13 (100)	
No	1 (2.3)	0 (0.0)	1 (4.0)		1 (3.2)	0 (0.0)	
Frequency of aerosolized antimicrobial prescription/recommendation **				0.166			0.919
At least once in three months	15 (34.1)	7 (36.8)	8 (32.0)		11 (35.5)	4 (30.8)	
At least once in six months	10 (22.7)	4 (21.1)	6 (24.0)		6 (19.4)	4 (30.8)	
At least once a month	8 (18.2)	1 (5.3)	7 (28.0)		6 (19.4)	2 (15.4)	
At least once a year	8 (18.2)	6 (31.6)	2 (8.0)		5 (16.1)	3 (23.1)	
At least once a week	1 (2.3)	0 (0.0)	1 (4.0)		1 (3.2)	0 (0.0)	
Indications for aerosolized antimicrobial agents **							
Treatment of bacterial infections	38 (86.4)	15 (78.9)	23 (92.0)	0.378	27 (87.1)	11 (84.6)	1.000
Prophylaxis against bacterial infections	17 (38.6)	11 (57.9)	6 (24.0)	**0.031** *	10 (32.3)	7 (53.8)	0.309 *
Treatment of fungal infections	3 (6.8)	2 (10.5)	1 (4.0)	0.570	1 (3.2)	2 (15.4)	0.204 *
Prophylaxis against fungal infections	7 (15.9)	5 (26.3)	2 (8.0)	0.210	2 (6.5)	5 (38.5)	**0.017** *
Treatment of viral infections	3 (6.8)	1 (5.3)	2 (8.0)	1.000	2 (6.5)	1 (7.7)	1.000
Prophylaxis against viral infections	1 (2.3)	0 (0.0)	1 (4.0)	1.000	1 (3.2)	0 (0.0)	1.000
Infection/diseases treated with aerosolized antimicrobial agents **							
Ventilator-associated pneumonia	30 (68.2)	10 (52.6)	20 (80.0)	0.101 *	22 (71.0)	8 (61.5)	0.724 *
Cystic fibrosis	27 (61.4)	17 (89.5)	10 (40.0)	**0.001** *	17 (54.8)	10 (76.9)	0.198 *
Ventilator-associated tracheobronchitis	15 (34.1)	3 (15.8)	12 (48.0)	0.052 *	13 (41.9)	2 (15.4)	0.162
Hospital-acquired pneumonia	13 (29.5)	5 (26.3)	8 (32.0)	0.749 *	9 (29.0)	4 (30.8)	1.000
Bronchiectasis	12 (27.3)	7 (36.8)	5 (20.0)	0.308 *	8 (25.8)	4 (30.8)	0.727
Others	2 (4.6)	2 (10.6)	0 (0.0)	0.181	1 (3.2)	1 (7.7)	0.508
Aerosolized antimicrobial agents prescribed/recommended							
Gentamicin	39 (88.6)	15 (78.9)	24 (96.0)	0.149	28 (90.3)	11 (84.6)	0.623
Colistin	32 (72.7)	14 (73.7)	18 (72.0)	1.000 *	22 (71.0)	10 (76.9)	1.000
Amikacin	11 (25.0)	4 (21.1)	7 (28.0)	0.731	6 (19.4)	5 (38.5)	0.256
Tobramycin	7 (15.9)	4 (21.1)	3 (12.0)	0.443	5 (16.1)	2 (15.4)	1.000
Amphotericin B	4 (9.1)	3 (15.8)	1 (4.0)	0.300	1 (3.2)	3 (23.1)	0.071
Others	3 (6.8)	2 (10.6)	1 (4.0)	0.570	2 (6.5)	1 (7.7)	1.000

** Rate calculated using item-specific denominator; * denotes Chi-Square Test; other analyses were performed using Fisher’s Exact Test; bold font denotes statistical significance.

**Table 3:** Treatment and administration strategies for aerosolized antimicrobial therapy among respondents.

	Total	Frequency (%)			Frequency (%)		
		Specialty			Profession		
Variable	Frequency (N = 44)	ID (N = 19)	Critical Care (N = 25)	*p* Value	Physicians (N = 31)	Pharmacists (N = 13)	*p* Value
Treatment approach				1.000			1.000
Aerosolized plus systemic antimicrobials	39 (88.6)	17 (89.5)	22 (86.0)		29 (93.5)	10 (76.9)	
Aerosolized antimicrobials alone	3 (6.8)	1 (5.3)	2 (8.0)		2 (6.5)	1 (7.7)	
Others	2 (4.5)	1 (5.3)	1 (4.0)		0 (0.0)	2 (15.4)	
Combination therapy strategy				0.519			**0.012**
Combine different antimicrobials for systemic and aerosolized administrations	29 (65.9)	14 (73.7)	15 (60.0)		21 (67.7)	8 (61.5)	
Do not combine aerosolized antimicrobial with other routes	5 (11.4)	1 (5.3)	4 (16.0)		3 (9.7)	2 (15.4)	
Combine the same antimicrobials for systemic and aerosolized administrations	7 (15.9)	2 (10.5)	5 (20.0)		7 (22.6)	0 (0.0)	
Others	3 (6.8)	2 (10.5)	1 (4.0)		0 (0.0)	3 (23.1)	
Microorganisms treated with aerosolized antimicrobial agents							
*Pseudomonas aeruginosa*	43 (97.7)	19 (100)	24 (96.0)	1.000	30 (96.8)	13 (100)	1.000
*Acinetobacter baumannii*	25 (56.8)	11 (57.9)	14 (56.0)	1.000 *	16 (51.6)	9 (69.2)	0.335 *
Enterobacterales	5 (11.4)	3 (15.8)	2 (8.0)	0.638	4 (12.9)	1 (7.7)	1.000
Staphylococcus species	2 (4.5)	1 (5.3)	1 (4.0)	1.000	1 (3.2)	1 (7.7)	0.508
Enterococcus species	2 (4.5)	0 (0.0)	2 (8.0)	0.498	2 (6.5)	0 (0.0)	1.000
Candida species	3 (6.8)	2 (10.5)	1 (4.0)	0.570	1 (3.2)	2 (15.4)	0.204
Does multidrug resistance or carbapenem resistance influence your recommendations for aerosolized antimicrobial agents?				0.557			0.823
It influences my recommendation to a great extent	11 (25.0)	4 (21.1)	7 (28.0)		7 (22.6)	4 (30.8)	
It influences my recommendation to some extent	24 (54.5)	12 (63.2)	12 (48.0)		17 (54.8)	7 (53.8)	
It does not influence my recommendation	9 (20.5)	3 (15.8)	6 (24.0)		7 (22.6)	2 (15.4)	
Administration device used for aerosolized antimicrobial therapy							
Jet nebulizer	14 (31.8)	5 (26.3)	9 (36.0)	0.495 *	11 (35.5)	3 (23.1)	0.498
Ultrasonic nebulizer	8 (18.2)	1 (5.3)	7 (28.0)	0.111	7 (22.6)	1 (7.7)	0.402
Vibrating mesh nebulizer	7 (15.9)	1 (5.3)	6 (24.0)	0.119	5 (16.1)	2 (15.4)	1.000
I do not know	19 (43.2)	14 (73.7)	5 (20.0)	0.001 *	11 (35.5)	8 (61.5)	0.182 *
Pre-treatment used prior to aerosolized antimicrobial therapy							
Bronchodilators	17 (38.6)	9 (47.4)	8 (32.0)	0.359 *	11 (35.5)	6 (46.2)	0.735 *
No pre-treatment	27 (61.4)	10 (52.6)	17 (68.0)	0.359 *	20 (64.5)	7 (53.8)	0.735 *
Patient factors that influence clinician decision to use aerosolized antimicrobial agents for respiratory tract infections							
Immunosuppression	32 (72.7)	13 (68.4)	19 (76.0)	0.735 *	24 (77.4)	8 (61.5)	0.295
Neutropenia	21 (47.7)	7 (36.8)	14 (56.0)	0.239 *	17 (54.8)	4 (30.8)	0.194 *
Age > 65 years	20 (45.5)	10 (52.6)	10 (40.0)	0.543 *	13 (41.9)	7 (53.8)	0.522 *
Chronic kidney disease	19 (43.2)	7 (36.8)	12 (48.0)	0.547 *	13 (41.9)	6 (46.2)	1.000 *
Solid organ transplant	15 (34.1)	4 (21.1)	11 (44.0)	0.199 *	11 (35.5)	4 (30.8)	1.000
Malignancy	13 (29.5)	3 (15.8)	10 (40.0)	0.105 *	10 (32.3)	3 (23.1)	0.722
Diabetes mellitus	10 (22.7)	1 (5.3)	9 (36.0)	**0.027**	8 (25.8)	2 (15.4)	0.697
Others	3 (6.8)	1 (5.3)	2 (8.0)	1.000	2 (6.5)	1 (7.7)	1.000
Adverse effects experienced by patients who received aerosolized antimicrobial therapy							
Bronchospasm	35 (79.5)	15 (78.9)	20 (80.0)	1.000	24 (77.4)	11 (84.6)	0.703
Hypoxemia	9 (20.5)	4 (21.1)	5 (20.0)	1.000	6 (19.4)	3 (23.1)	1.000
Others	3 (6.8)	2 (10.5)	1 (4.0)	0.570	3 (9.7)	0 (0.0)	0.544
No adverse effect	7 (15.9)	3 (15.8)	4 (16.0)	1.000	5 (16.1)	2 (15.4)	1.000
Have a hospital protocol for aerosolized antimicrobial therapy				0.421			0.490
Yes	8 (18.2)	5 (26.3)	3 (12.0)		7 (22.6)	1 (7.7)	
No	25 (56.8)	9 (47.4)	16 (64.0)		16 (51.6)	9 (69.2)	
I do not know	11 (25.0)	5 (26.3)	6 (24.0)		8 (25.8)	3 (23.1)	

* Denotes Chi-Square Test; other analyses were performed using Fisher’s Exact Test; bold font denotes statistical significance.

**Table 4:** Clinicians’ perceptions towards aerosolized antimicrobial therapy.

	Total	Frequency (%)			Frequency (%)		
		Specialty			Profession		
Variable	Frequency (N = 44)	ID (N = 19)	Critical Care (N = 25)	*p* Value	Physicians (N = 31)	Pharmacists (N = 13)	*p* Value
How well informed are you about aerosolized antimicrobial therapy?				**0.031**			0.162
Very informed	2 (4.5)	0 (0.0)	2 (8.0)		1 (3.2)	1 (7.7)	
Well informed	14 (31.8)	10 (52.6)	4 (16.0)		8 (25.8)	6 (46.2)	
Neutral	21 (47.7)	8 (42.1)	13 (52.0)		15 (48.4)	6 (46.2)	
Not well informed	7 (15.9)	1 (5.3)	6 (24.0)		7 (22.6)	0 (0.0)	
Rating the need for training on aerosolized antimicrobial therapy				0.345			0.379
1	1 (2.3)	0 (0.0)	1 (4.0)		0 (0.0)	1 (7.7)	
2	7 (15.9)	3 (15.8)	4 (16.0)		4 (12.9)	3 (23.1)	
3	16 (36.4)	5 (26.3)	11 (44.0)		12 (38.7)	4 (30.8)	
4	16 (36.4)	10 (52.6)	6 (24.0)		11 (35.5)	5 (38.5)	
5	4 (9.1)	1 (5.3)	3 (12.0)		4 (12.9)	0 (0.0)	
Aerosolized antimicrobial therapy improves clinical cure				0.294			**0.015**
Strongly agree	4 (9.1)	1 (5.3)	3 (12.0)		4 (12.9)	0 (0.0)	
Agree	14 (31.8)	4 (21.1)	10 (40.0)		12 (38.7)	2 (15.4)	
Neutral	18 (40.9)	8 (42.1)	10 (40.0)		13 (41.9)	5 (38.5)	
Disagree	5 (11.4)	4 (21.1)	1 (4.0)		2 (6.5)	3 (23.1)	
Strongly disagree	3 (6.8)	2 (10.5)	1 (4.0)		0 (0.0)	3 (23.1)	
Aerosolized antimicrobial therapy improves microbial cure				**0.029**			**0.016**
Strongly agree	4 (9.1)	8 (42.1)	4 (16.0)		4 (12.9)	0 (0.0)	
Agree	14 (31.8)	0 (0.0)	6 (24.0)		8 (25.8)	6 (46.2)	
Neutral	18 (40.9)	5 (26.3)	13 (32.0)		16 (51.6)	2 (15.4)	
Disagree	6 (13.6)	5 (26.3)	1 (4.0)		3 (9.7)	3 (23.1)	
Strongly disagree	2 (4.5)	1 (5.3)	1 (4.0)		0 (0.0)	2 (15.4)	
Aerosolized antimicrobial therapy reduces systemic toxicity				0.197			0.497
Strongly agree	6 (13.6)	2 (10.5)	4 (16.0)		5 (16.1)	1 (7.7)	
Agree	30 (68.2)	15 (78.9)	15 (60.0)		21 (67.7)	9 (69.2)	
Neutral	7 (15.9)	1 (5.3)	6 (24.0)		5 (16.1)	2 (15.4)	
Strongly disagree	1 (2.3)	1 (5.3)	0 (0.0)		0 (0.0)	1 (7.7)	
Aerosolized antimicrobial therapy allows for the administration of higher doses of antimicrobial agents				0.213			**0.045**
Strongly agree	1 (2.3)	0 (0.0)	1 (4.0)		1 (3.2)	0 (0.0)	
Agree	15 (34.1)	8 (42.1)	7 (28.0)		8 (25.8)	7 (53.8)	
Neutral	15 (34.1)	4 (21.1)	11 (44.0)		13 (41.9)	2 (15.4)	
Disagree	11 (25.0)	5 (26.3)	6 (24.0)		9 (29.0)	2 (15.4)	
Strongly disagree	2 (4.5)	2 (10.5)	0 (0.0)		0 (0.0)	2 (15.4)	
Aerosolized antimicrobial therapy can reduce antimicrobial resistance				0.592			0.140
Strongly agree	2 (4.5)	0 (0.0)	2 (8.0)		2 (6.5)	0 (0.0)	
Agree	11 (25.0)	6 (31.6)	5 (20.0)		7 (22.6)	4 (30.8)	
Neutral	18 (40.9)	6 (31.6)	12 (60.0)		15 (48.4)	3 (23.1)	
Disagree	11 (25.0)	6 (31.6)	5 (20.0)		7 (22.6)	4 (30.8)	
Strongly disagree	2 (4.5)	1 (5.2)	1 (4.0)		0 (0.0)	2 (15.4)	
Barriers to the use of aerosolized antimicrobial therapy for respiratory infections **							
Limited evidence on efficacy	39 (88.6)	18 (94.7)	21 (84.0)	0.370	26 (63.9)	13 (100)	0.301
Availability of medication	15 (34.1)	6 (31.6)	9 (36.0)	1.000 *	10 (32.3)	5 (38.5)	0.737
Limited evidence on safety	9 (20.5)	4 (21.1)	5 (20.0)	1.000	4 (12.9)	5 (38.5)	0.098
Cost of therapy	6 (13.6)	1 (5.3)	5 (20.0)	0.213	5 (16.1)	1 (7.7)	0.652
Availability of administration device	1 (2.3)	1 (5.3)	0 (0.0)	0.432	1 (3.2)	0 (0.0)	1.000

** Rate calculated using item-specific denominator; * denotes Chi-Square Test; other analyses were performed using Fisher Exact Test; bold font denotes statistical significance.

## Data Availability

The datasets generated and analyzed during the current study are not publicly available due to institutional privacy policies, but are available from the corresponding author on reasonable request.
